# Cell Size Critically Determines Initial Retention of Bone Marrow Mononuclear Cells in the Heart after Intracoronary Injection: Evidence from a Rat Model

**DOI:** 10.1371/journal.pone.0158232

**Published:** 2016-07-05

**Authors:** Niall G. Campbell, Masahiro Kaneko, Yasunori Shintani, Takuya Narita, Vinit Sawhney, Steven R. Coppen, Kenta Yashiro, Anthony Mathur, Ken Suzuki

**Affiliations:** William Harvey Research Institute, Barts and the London School of Medicine and Dentistry, Queen Mary University of London, London, United Kingdom; French Blood Institute, FRANCE

## Abstract

Intracoronary injection of bone marrow mononuclear cells (BMMNC) is an emerging treatment for heart failure. Initial donor cell retention in the heart is the key to the success of this approach, but this process remains insufficiently characterized. Although it is assumed that cell size of injected cells may influence their initial retention, no scientific evidence has been reported. We developed a unique model utilizing an *ex-vivo* rat heart perfusion system, enabling quantitative assessment of retention of donor cells after intracoronary injection. The initial (5 minutes after intracoronary injection) retention rate of BMMNC was as low as approximately 20% irrespective of donor cell doses injected (1×10^6^, 8×10^6^, 4×10^7^). Quantitative cell-size assessment revealed a positive relationship between the size of BMMNC and retention ratio; larger subpopulations of BMMNC were more preferentially retained compared to smaller ones. Furthermore, a larger cell type—bone marrow-derived mesenchymal stromal cells (median size = 11.5μm *versus* 7.0μm for BMMNC)—had a markedly increased retention rate (77.5±1.8%). A positive relationship between the cell size and retention ratio was also seen in mesenchymal stromal cells. Flow-cytometric studies showed expression of cell-surface proteins, including integrins and selectin-ligands, was unchanged between pre-injection BMMNC and those exited from the heart, suggesting that biochemical interaction between donor cells and host coronary endothelium is not critical for BMMNC retention. Histological analyses showed that retained BMMNC and mesenchymal stromal cells were entrapped in the coronary vasculature and did not extravasate by 60 minutes after transplantation. Whilst BMMNC did not change coronary flow after intracoronary injection, mesenchymal stromal cells reduced it, suggesting coronary embolism, which was supported by the histological finding of intravascular cell-clump formation. These data indicate that cell-size dependent, passive (mechanical), intravascular entrapment is responsible for the initial donor cell retention after intracoronary injection of BMMNC in the heart having normal vasculatures (at least).

## Introduction

Transplantation of unfractionated bone marrow mononuclear cells (BMMNC) *via* intracoronary (IC) injection is a promising approach for the treatment of not only acute myocardial infarction but also chronic heart failure [1–6]. IC injection has been reported to have advantages, as a cell-delivery route for stem cell transplantation to the heart, over other current methods, including transendocardial intramyocardial injection, while there are controversial reports [7–9]. Either way, following encouraging pre-clinical studies, randomized clinical trials have reported that IC injection of BMMNC leads to improvements in cardiac function, quality of life and survival in patients with ischemic and non-ischemic dilated cardiomyopathy. The degree of the therapeutic effects observed in previous clinical trials was, however, not satisfactory, and also there are negative reports [10,11], proposing the requisition of further understanding and refinement of the protocols for BMMNC-based therapy to be widely established [12,13]. One important reason associated with this treatment is poor ‘engraftment’ of BMMNC in the recipient heart after transplantation [14–16]. Engraftment of donor cells after IC injection is the consequence of a number of donor cell behaviors, including initial retention, trans-endothelial migration into myocardial interstitium (or integration into vascular walls) and survival with/without differentiation. Among these processes, initial retention has been suggested to be the major determinant of successful engraftment of transplanted cells *via* IC injection [15,16]. In a porcine study that dynamically tracked radiolabelled BMMNC after IC injection, it was shown that appropriately 80% of cells were flushed out of the heart within 2 minutes of injection [17]. Initial retention could theoretically encompass the processes of “active (biochemical)” adhesion of donor cells to the coronary endothelium *via* adhesion molecules and integrins, or/and “passive (mechanical)” entrapment in the intravascular lumen [18]. However, our understanding of the mechanism responsible for the initial donor cell retention remains insufficient.

There are a limited number of available models to investigate initial donor cell retention after IC injection in a quantitative manner. The most frequent method used for this purpose is transplantation of radiolabelled cells *via* a catheter inserted into the coronary artery, followed by measurement of radioactivity of the heart, either in large animals [17–19] or human subjects [20,21]. However, these models do not enable collection of donor cells retained in or exited from the heart after IC injection, which would allow characterization of these cells to obtain important information on initial retention of donor cells. In addition, using these current methods, it is difficult to compare donor cell retention between various treatment protocols (*i*.*e*. cell types or cell numbers) and to assess minute-by-minute donor cell retention within the initial few minutes of IC injection, due to demanding technical and financial burden.

We have reported a model using *ex-vivo* Langendorff perfusion of a mouse heart, which is capable of assessing quantitative donor cell retention after IC injection [16]. In this study, we further advanced this original model for use in rats. Because it is much easier to establish a reproducible Langendorff heart perfusion model in rats compared to mice, the development of a rat model will generate a more user-friendly, generic experimental technique. We could also inject larger numbers of cells into the coronary artery in a rat, compared to a mouse, allowing more precise measurements of donor cell retention. Using such a rat model, we investigated BMMNC retention on a minute-by-minute basis immediately following IC injection of different numbers of BMMNC (also in comparison with another cell-type, mesenchymal stromal cells [MSC]) in a quantitative manner. Furthermore, by comparing characteristics between donor cells before injection and those flushed away into coronary effluent, this approach enabled the underlying mechanisms of early retention to be studied.

## Materials and Methods

### Ethical approval of animal studies

This study was carried out in strict accordance with the recommendations in the Guide for the Care and Use of Laboratory Animals of the National Institutes of Health. All animal protocols were approved by the ethics committee at the Queen Mary University of London and the UK Home Office (Project License; PPL70/7254). All animal procedures were carried out only by UK Home Office Personal License holders, and all efforts were made to minimize suffering.

### BMMNC preparation

BMMNC were isolated from male Sprague-Dawley rats (200–250 g body weight; Charles River, UK) as described previously [16,22]. Rats were sacrificed by cervical dislocation following isofluorane anesthesia (Abbot) using an inhalation anesthesia unit (Vet Tech Solutions Ltd with Fluorovac Harvard Solutions). The femurs and tibias were dissected, carefully cleaned of adherent soft tissue and transported in sterile PBS on ice. The epiphyses were removed with a rongeur, and the marrow was harvested by inserting a 19-gauge syringe needle into one end of the bone whilst flushing with PBS into a 50 ml tube. Then, the bones were cut longitudinally using a rongeur and adherent bone marrow tissue was flushed with PBS into the tube. The 50 ml tube was centrifuged at 400 g for 5 minutes and the supernatant was removed. The bone marrow cell pellet was resuspended in PBS, filtered through a 100-μm filter and centrifuged at 400 *g* for 5 minutes. The supernatant was removed, and the bone marrow cells were resuspended in 5 ml PBS. The cell suspension was then carefully layered by pipette onto 7 ml Ficoll (Sigma) and subjected to gradient centrifugation at 1040 g for 30 minutes. Cells at the interface were collected as BMMNC and washed in PBS. Viability of collected BMMNC before injection was always >94%.

### MSC preparation

Bone marrow-derived MSC were isolated from male Sprague-Dawley rats (200 g body weight; Charles River, UK) as described previously [23]. Whole bone marrow cells were isolated as described above, excluding the gradient centrifugation steps. Collected whole bone marrow cells were placed in 25cm^2^ flasks (Nunc) with an initial plating concentration of approximately 1×10^6^ cells/cm^2^, cultured in αMEM (Gibco) with 20% inactivated fetal bovine serum containing 200 mM L-glutamine, 100 U/ml penicillin and 100 mg/ml streptomycin (Sigma) at 37°C in a humidified atmosphere containing 95% air / 5% CO_2_. The culture medium was changed every 48–72 hours. When cell confluence reached 80%, cells were passaged by using 0.25% Trypsin / 0.2% EDTA (Sigma). Plating concentrations for subsequent passages were 2.5×10^4^ cells/cm^2^. MSC were used for experiments at passage 4 or 5. Viability of collected MSC before injection was always >96%.

### Cell number counting and cell size analysis

An automated cell counter (Countess Automated Cell Counter, Invitrogen) was used to quantify the cell number and cell-size distribution of cell populations. This desktop device quantifies the number of cells of each diameter, according to 1-μm subdivisions, as well as the number of dead cells using Trypan blue. The mean cell counts and cell-size distribution for each sample was acquired from at least 3 measurements.

### Donor cell labeling

For identification of donor cells in the host heart by histological analyses, donor cells were labeled by PKH67 (Sigma), a green fluorescent dye, before IC injection according to the manufacturer’s recommendations (10 minutes incubation with 2 μM PKH67 at room temperature). This resulted in high staining efficiency (>99%) with a low proportion of non-viable cells (<2%).

### Perfusion of the rat heart using a Langendorff unit

Male Sprague-Dawley (200 g body weight) rats were purchased from Charles River, UK, anaesthetized with isoflurane inhalation (Abbott, USA), and administered with heparin (100 iU/kg; Leo Pharma, FL, USA) *via* intraperitoneal injection. After 5 minutes, cervical neck dislocation was performed, and a median sternotomy was performed. The heart was excised, placed immediately in ice-cold modified Krebs-Henseleit buffer [16] (NaCl 120 mM, KCl 4.5 mM, MgSO_4_ 1.2 mM, KH_2_PO_4_ 1.2 mM, CaCl_2_ 2 mM, NaHCO_3_ 20 mM, Glucose 10.0 mM in 2L ddH_2_O, gassed with continuous 95% O_2_ /5% CO_2_) and taken for immediate cannulation. A careful incision of the aorta was made at the level of the aortic arch enabling the ascending aorta to be cannulated with a blunt ended 14-gauge needle (Harvard). The aorta was temporarily clipped to the cannula and, once appropriate perfusion was confirmed, the aorta was secured to the cannula with a 5–0 silk suture (Bear, Japan). A small cut was made in the proximal pulmonary artery to ensure that coronary effluent could drain freely. The heart was perfused by the warmed (37°C), modified Krebs-Henseleit Buffer at a constant pressure manually maintained at 95–105 cmH_2_O. By 20 minutes after re-perfusion, the hearts were usually stably perfused by exhibiting stable perfusion parameters such as coronary flow, heart rate, and regular sinus rhythm without arrhythmias.

### IC cell injection into Langendorff-perfused rat hearts

At 20 minutes after the start of *ex-vivo* heart perfusion, a designated number of donor cells (either BMMNC or MSC) suspended in 3 ml PBS were injected into the heart from a side port (3-way tap) of the aortic cannula using a 5 ml syringe. We have conformed that donor cell loss due to dead space or adhesion to the walls of syringe/tubes/glassware was negligible (<1% of the total volume injected). Cell injection was slowly performed over 20 seconds to prevent donor cells from high pressure. During injection, the aortic valve remains physiologically closed, and thereby all cells entered the coronary arteries.

### Choice of donor cell number injected

It was important for the project to utilize a range of cell doses that replicate those used in clinical trials. In previous clinical trials of IC injection of BMMNC [1–13], the cell number IC injected typically ranged from 1×10^8^ to 1×10^10^. In contrast to our model where cells were injected into all the major coronary arteries (right, left anterior descending and left circumflex), clinical trials usually injected the cells into a single coronary artery. The assumption was made that the median cell number used in clinical trials was 1×10^9^ BMMNC for a 75 kg human and that a rat’s weight is 200 g. Therefore the equivalent dose for a single rat artery would be 2.7×10^6^ BMMNC, or 8×10^6^ BMMNC injected into all three arteries. Based on this calculation, we decided to investigate the 3 BMMNC numbers; 1×10^6^, 8×10^6^ and 40×10^6^. As regards MSC, in order to compare with BMMNC, 1×10^6^ cells were injected.

### Coronary effluent cell counting and calculation of retention rate

“Coronary Effluent Cell Concentration” (cells/ml) in each collected coronary effluent was measured using a Countess Automated Cell Counter (Invitrogen, UK). The coronary effluent flow rate (ml/min) was noted and utilized to calculate the “Coronary Effluent Cell Number” (cells/min) at each minute. All measurements were made in triplicate. **“**Total Donor Cell Number in Coronary Effluent**”** was then calculated as the sum of **“**Coronary Effluent Cell Number**”** over the first 5 minutes, as it was confirmed that almost 100% of BMMNC leakage from the normal heart occurred within 5 minutes. Then, **“**Donor Cell Retention Rate (%)**”** was calculated as (“Injected Donor Cell Number”–“Total Donor Cell Number in Coronary Effluent”) ÷ “Injected Donor Cell Number” ×100.

There was a significant leakage of red blood cells into the coronary effluent in the initial minutes after the start of *ex-vivo* heart perfusion. However, the number of red blood cells counted in the coronary effluent after 20 minutes was low enough for the project’s purposes (<1x10^5^/min), at which time donor cells were injected.

### Flow cytometric study

Flow cytometry was performed to determine the expression of relevant cell-surface proteins on isolated BMMNC prior to cell injection and on donor cells in the coronary effluent (collected over 5 minutes after IC injection of 8x10^6^ BMMNC). For each analysis, at least 1×10^6^ BMMNC were collected and suspended in Flow Cytometry Buffer (HBSS, 0.025% bovine serum albumin, 2 mM EDTA) and placed in separate tubes. Relevant primary antibody was added and incubated on ice for 30 minutes. Primary antibodies used were: CD11b (BD Phamingen, mouse anti-rat monoclonal, 5 μg/ml); CD18 (BD Pharmingen, mouse anti-rat monoclonal, 5 μg/ml); CD31 (BD Pharmingen, mouse anti-rat monoclonal, 5 μg/ml); CD34 (Santa Cruz, USA, mouse anti-rat monoclonal, 10 μg/ml); CD44 (AbD Serotec, mouse anti-rat monoclonal, 10 μg/ml); CD45 (BD Pharmingen, mouse anti-rat monoclonal, 5 μg/ml); CD90: (BD Pharmingen, mouse anti-rat monoclonal, 5 μg/ml); and CD162 (Bios, rabbit anti-human polyclonal but cross react to the rat antigen, 10 μg/ml). After washing, to minimize background signal from secondary antibody administration, samples were treated with 5 μg/ml anti rat FAB antibody (Polyclonal goat FAB anti rat Ig (H+L), Southern Biotech, USA) for 30 minutes, followed by further washing, before secondary antibody administration (goat anti-mouse IgG (H+L) Invitrogen) and incubation on ice for a further 30 minutes. Mouse IgAk and mouse IgGk1 isotype controls (BD Pharmingen, 5 μg/ml) were used as control primary antibodies. For analysis of CD29, Alexa Fluor 647-conjugated hamster anti-rat antibody (Biolegend, USA, 5 μg/ml) was incubated. Alexa-Fluor 647-IgG isotype control (ebioscience, 5 μg/ml) was used as a control antibody. Analysis was then performed on a DAKOcyan Flow Cytometry Machine using Summit 4.3 software. For data analysis, cells were included in the analysis if they were viable (based on propidium iodide staining) and within the main population of cells based on forward and side scatter analysis. The proportion of viable cells in all the staining protocols was >90%. Voltage gating was determined from the relevant negative controls.

### Histological examination

At a chosen time, Langendorff-perfused hearts were cut and incubated in 4% ice-cold PFA for 1 hour, followed by overnight incubation in 30% sucrose at 4°C. The hearts were then frozen with OCT compound using liquid nitrogen, from which 7.5 μm transverse cryosections were cut. The sections were blocked with 10% goat serum for 60 minutes and then incubated with 1/100 Biotinylated *Griffonia* (*Bandeiraea*) *Simplicifolia* Lectin I Isolectin B4 antibody (Vector laboratories, 0.5mg/ml) in 5% goat serum for 60 minutes. Isolectin-B4 is a marker for endothelial cells [20]. After washing, sections were incubated with 1/400 dilution of Streptavidin—Alexa 594 (Invitrogen, UK, 2 mg/ml, Catalogue number s32356), together with 1/1000 dilution of DAPI (Roche, Catalogue: 10236276001) for nuclear counterstaining, for a further 60 minutes. The sections were observed using fluorescent microscopy (BZ7000, Keyence) with DAPI-B, GFP and TxRed filters (Keyence).

### Estimation of donor cell retention from histological data

At five minutes after IC injection of 8x10^6^ PKH67-labeled BMMNC, the heart (n = 4) was fixed and frozen as described above. From these, three 7.5 μm-thick horizontal cryosections (one each from the center [papillary muscle level], upper (base) and lowers (apex) parts of the heart) were produced. The total number of PKH67^+^ cells was counted throughout each cryosection, divided by the myocardial area studied (measured using Image J), and averaged in each heart. Then, for estimation of donor BMMNC number retained in the whole heart, these obtained averaged PKH67^+^ cell numbers (cells/mm^2^) were exchanged to the PKH67^+^ cell density (cells/mm^3^) based on the fact that the thickness of the cryosection was 7.5 μm. The myocardial volume was estimated from the heart weight (1,242±21 mg), with assumption that 1 mm^3^ rat heart tissue is 1 mg. Thus, the number of retained BMMNC in the whole heart was calculated with the formula:

$$=\;[\text{averaged}\;\text{PKH}67^{+}\text{ cell}\;\text{number}\;(\text{cells}/\text{m}{\text{m}}^{2})]\;÷\;0.0075\;\text{x}\;\text{heart}\;\text{weight}\;\left(\text{mg}\right)$$

The retention rate was therefore calculated as:

$$=\;\left[\text{number}\;\text{of}\;\text{retained}\;\text{BMMNC}\;\text{in}\;\text{the}\;\text{whole}\;\text{heart}\right]\;/\;8\text{x}10^{6}\;\text{x}\;100\;\left(\%\right)$$

In addition, the numbers of PKH67^+^ cells in each of epicardial, myocardial or endocardial parts of the left ventricular wall were counted in each cryosection cut from the center [papillary muscle level], upper (base) and lowers (apex) parts of the heart, divided by the myocardial area studied (measured using Image J), and averaged in each area. Then the value in each area was averaged among 4 hearts.

### Statistical analysis

Normally distributed data was expressed as mean±standard error of the mean (SEM). Where data was not normally distributed, a median value was quoted. A *p* value <0.05 was pre-determined to represent statistical significance. GraphPad Prism 5.01 and SPSS12.0 were used for the purposes of statistical analysis. When comparing two sets of quantitative data, a paired or unpaired T-test (where appropriate) was utilized. When comparing more than two sets of quantitative data, a one-way or two-way (where relevant) ANOVA was utilized (the details are presented in each Figure Legend).

## Results

### Time course of donor cell loss, retention rate, and coronary flow after IC injection of BMMNC into the rat heart

**Fig 1:**
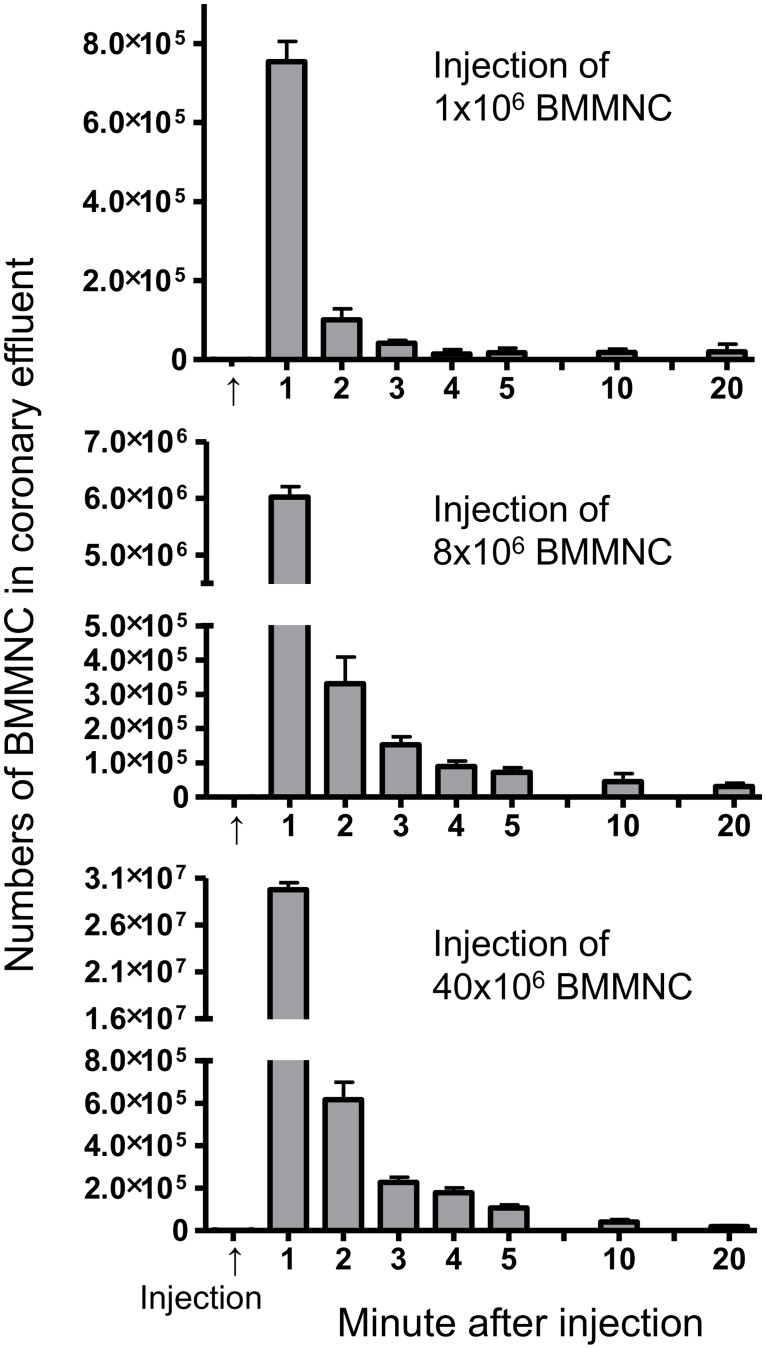
Exit of BMMNC from the heart after IC injection. After IC injection of three different doses (1, 8, 40×10^6^) of rat BMMNC in to rat hearts using the Langendorff *ex-vivo* perfusion model, the numbers of BMMNC in the coronary effluent in every minute were counted. The majority of BMMNC injected exited from the heart into coronary effluent in 1 minute after IC injection of any cell dose studied. The pattern of cells exited from the heart did not differ between the doses (n = 8 different hearts studied in each dose, *p>0*.*99*, Two-way repeated measure ANOVA). Upper panel for 1×10^6^ BMMNC injection; Middle panel for 8×10^6^ BMMNC injection; Lower panel for 40×10^6^ BMMNC injection.

**Fig 2:**
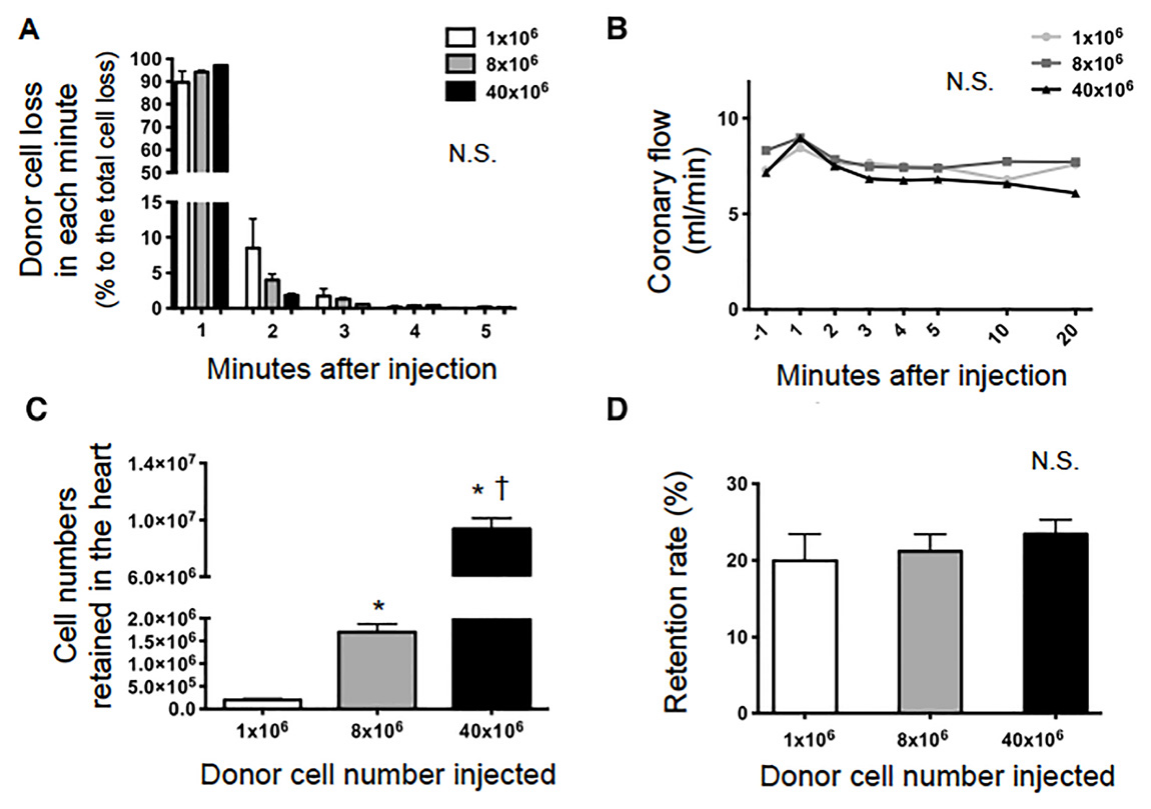
Quantitative analysis of initial retention of BMMNC after IC injection. (**A**) After IC injection of three different doses (1, 8, 40×10^6^) of rat BMMNC in to a rat heart, the numbers of BMMNC in the coronary effluent in every minute were counted (Fig 1), from which their proportions to the total BMMNC exited in the coronary effluent over the first 5 minutes were calculated. It was observed that over 90% of the cells that were lost into the coronary effluent exited the heart within the first minute of injection. The proportion of cells lost did not differ between the doses (n = 8 hearts studied in each dose, *p>0*.*99*, Two-way repeated measures ANOVA). (**B**) Coronary flow rate was not altered by BMMNC injection using these three doses (n = 8 in each dose, *p* = 0.22, Two-way repeated measures ANOVA). The slight increase in volume during the first minute was due to injection of 3 ml of cell suspension. **(C,D)** Absolute numbers and rates of donor BMMNC retained in the heart were calculated. A larger number of injected cells resulted in a larger number of retained cells in the hearts (**C**, n = 8 in each dose, *p*<0.0001, One-way ANOVA followed by Bonferroni post-hoc test. *p<0.05 versus 1x10^6^ group, ^†^p<0.05 versus 8x10^6^ group). On the other hand, the proportion of retained cells of the total injected cell numbers was unchanged among the three doses (**D**; n = 8 in each dose, *p* = 0.67, One-Way ANOVA).

Using our original rat model based on Langendorff *ex-vivo* heart perfusion, donor cell loss (exiting from the heart into the coronary effluent) was quantified minute-by-minute after IC injection of rat BMMNC (1×10^6^, 8×10^6^ or 40×10^6^ cells injected). The majority of donor cells were lost into coronary effluent immediately after IC injection (Fig 1). After 5 minuets of IC injection, exit of donor BMMNC into the coronary effluent hardly occurred. There was no difference in the general pattern of donor cell loss from the heart after IC injection between the 3 BMMNC doses. Calculation of these data clarified that more than 90% of the total cells that exited the heart were flushed away within the first minute of injection, regardless of BMMNC numbers injected (Fig 2A). Coronary effluent volumes exiting the heart (coronary flow) were serially measured before and after IC injection of BMMNC (Fig 2B). There was no decrease in coronary flow rates after BMMNC injection, demonstrating that myocardial perfusion was not impaired by IC injection of BMMNC with the range of cell doses studied. This suggested that significant coronary embolism did not occur after IC transplantation of up to 40×10^6^ BMMNC into the normal heart in rats.

By calculating above data of donor cell exist into the coronary effluent, it was found that a larger cell dose resulted in a proportionally higher number of donor BMMNC retained in the heart (Fig 2C), whilst the rate of initial retention (% retained cells in 5 minutes after IC injection to the donor cell dose injected) was approximately 20%, irrespective of initial donor cell doses (Fig 2D). This implies that a distinct subpopulation of injected BMMNC, which have specific cellular properties, might be preferentially retained in the heart after injection.

### Positive relationship between cell size and retention rate after IC injection of BMMNC

**Fig 3:**
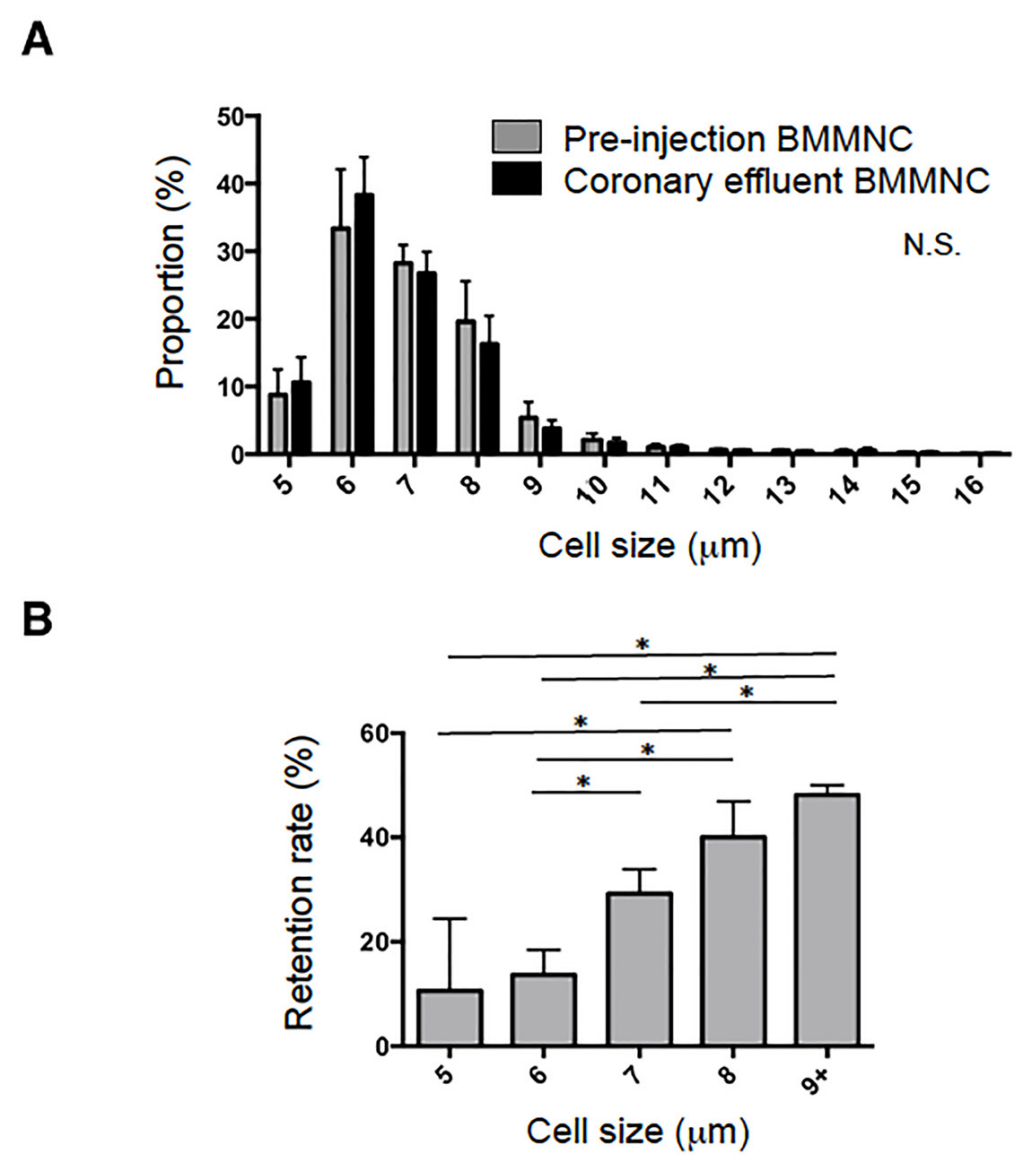
Cell size-dependent retention of BMMNC after IC injection. (**A**) Distributions of BMMNC diameters prior to injection and those in the coronary effluent after IC injection of 8×10^6^ BMMNC were quantified and expressed as fractions. There appeared to be a leftwards shift in the diameters of the coronary effluent cell population but this difference was not statistically significant (n = 8 different hearts in each cell size, *p* = 0.21, Two-way repeated measures ANOVA). (**B**) With knowledge of the cell numbers of each cell diameter, the retention rate of BMMNC having each diameter could be calculated. It was observed that larger donor BMMNC were increasingly likely to be retained (n = 8, *p* = 0.01, One-way ANOVA followed by Bonferroni post-hoc test *p<0.05).

To investigate the impact of cell size as a factor to affect initial retention, the distribution of the diameters of donor BMMNC prior to injection and that of BMMNC in the coronary effluent was compared. The mean and median sizes of BMMNC prior to injection were both 7.0 μm, and the population had a parabolic distribution with a leftwards skew (Fig 3A). Cells ≥10 μm in diameter accounted for less than 1% of the total BMMNC population. In contrast, the mean and median cell sizes of BMMNC in the coronary effluent were 6.6 and 7.0 μm, respectively. The coronary effluent cells appeared to exhibit a leftwards shift in cell size, with an increase in the proportion of smaller BMMNC and a reduction in the proportion of larger BMMNC, but this difference was not statistically significant (p = 0.21).

With knowledge of the size distributions of BMMNC prior to IC injection and BMMNC leaked into the coronary effluent, the retention rate of injected BMMNC of each cell-size subpopulation could be calculated. It was found that the retention rate increased progressively with larger cell diameters (Fig 3B). Whereas only <10% of BMMNC with a diameter of 5–6 μm were retained after IC injection, approximately 50% of BMMNC sized ≥9 μm were retained.

### No relationship between cell-surface protein expression and retention of BMMNC

**Fig 4:**
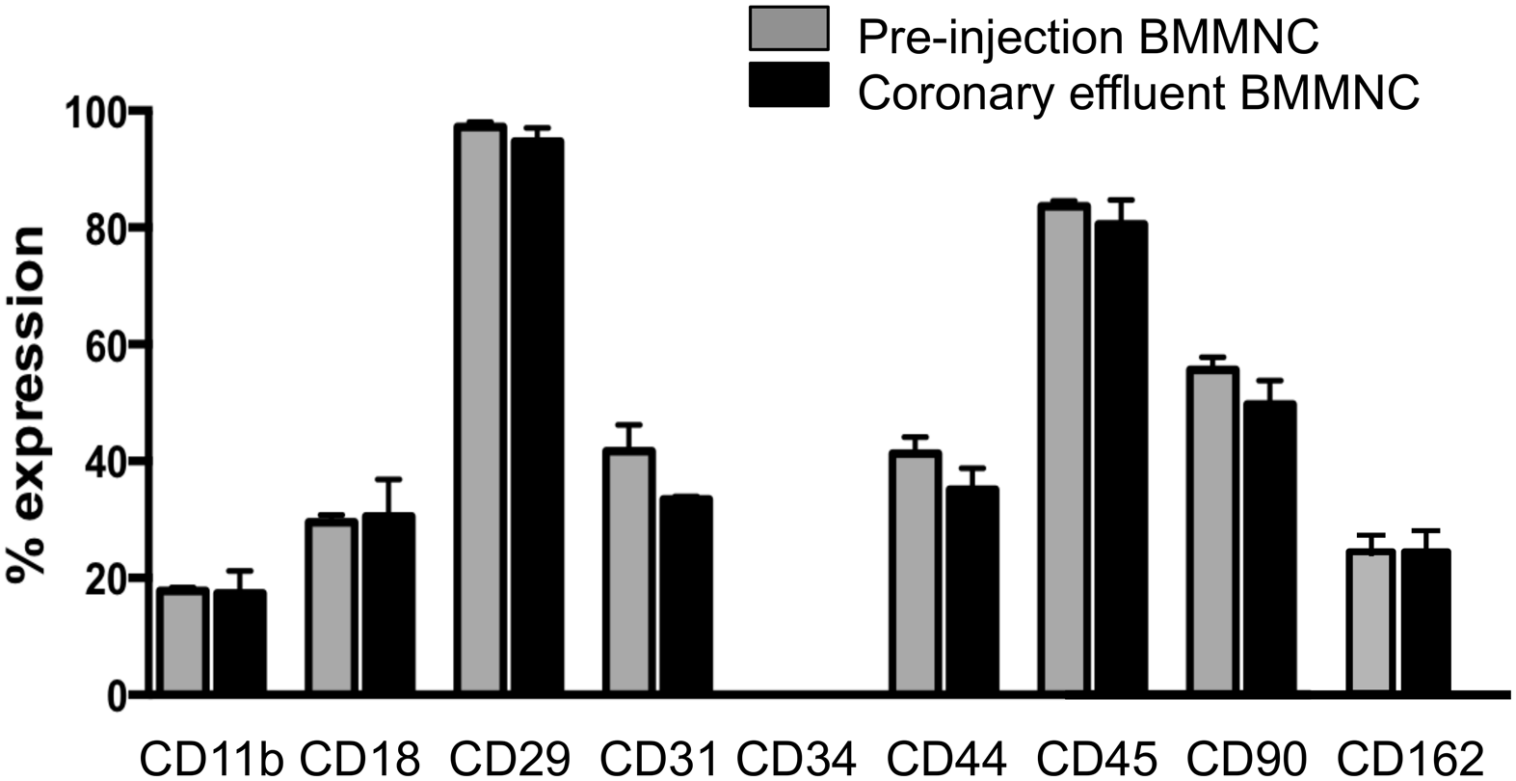
Relationship of cell-surface proteins and retention of BMMNC. The expression profiles of cell-surface proteins, including integrin and selectin ligand, on pre-injection BMMNC and BMMNC in the coronary effluent (collected over the five-minute duration post-IC injection of 8x10^6^ BMMNC) were compared by flow cytometric analysis. No difference was found in any of the surface proteins investigated (n = 6 hearts were studied, unpaired T-test), suggesting that these cell-surface proteins are not critical for retention in normal hearts.

It was hypothesized that specific subpopulations of BMMNC expressing certain specific cell-surface proteins, including cell adhesion-related molecules such as integrins, selectin ligands and adhesion molecules, may be preferentially retained in the heart after IC injection (= active, biochemical adhesion of BMMNC to the coronary endothelium). To study this, the profile of specific surface proteins expressed in pre-injection BMMNC and coronary effluent cells was determined and compared by flow cytometry (Fig 4). Cell-surface molecules studied included CD29 (integrin β1), CD18 (integrin β2), CD11b (integrin αM), CD162 (P-selectin glycoprotein ligand-1), PECAM-1 (CD31), CD34, CD44, CD45, and CD90. There was no difference in expression of any surface protein studied between the pre-injection and coronary effluent BMMNC, suggesting that these surface molecules do not play a role in BMMNC retention in the normal heart, and that biochemical interactions (active adhesions) between donor BMMNC and the coronary endothelium are not critical for retention in normal hearts.

### Histological assessment of BMMNC retention in the heart

**Fig 5:**
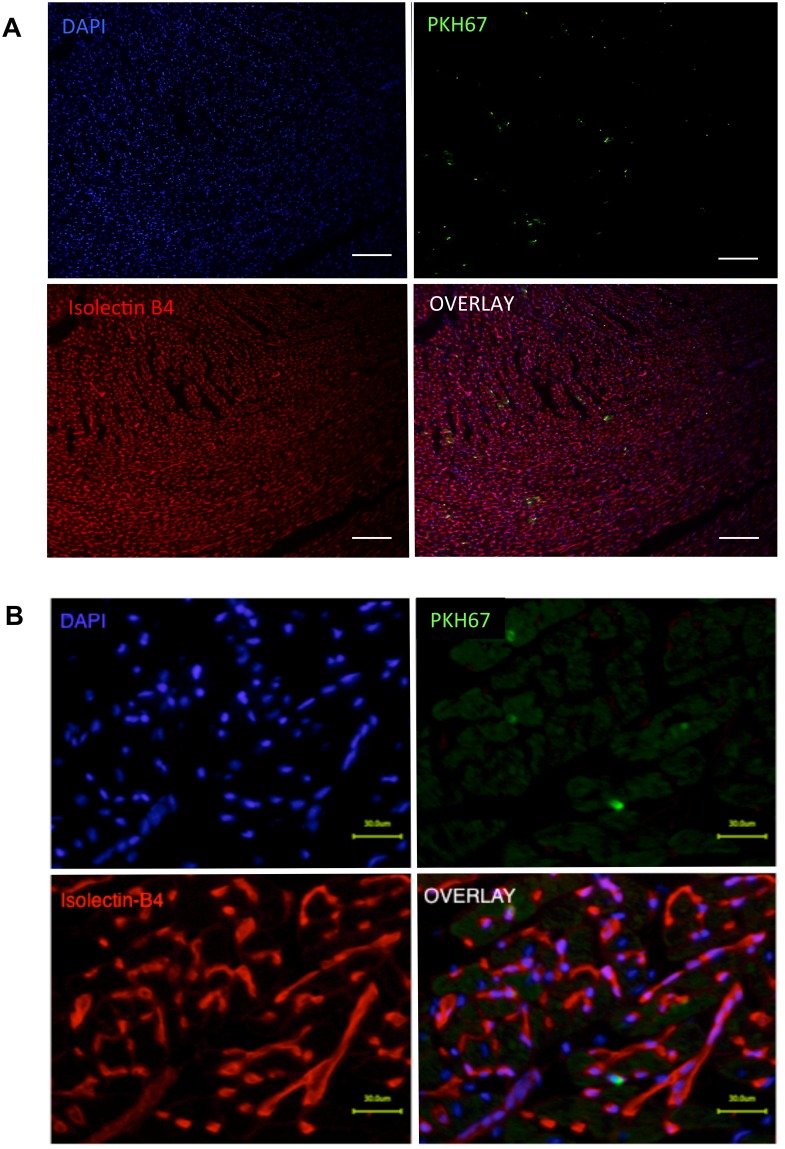
Histological findings of BMMNC retention 5 min after IC injection. Recipient hearts were collected at 5 minutes after IC injection of 8x10^6^ BMMNC that had been labeled with PKH67. **A** and **B** represent low- and high-magnification images of Isolectin-B4 staining, respectively (scale bar = 100 μm [A] and 30 μm [B]). Representative micro images from n = 4 hearts are presented. All donor BMMNC (green) retained in the heart were found to be entrapped in isolation and within the coronary microvasculature. Blue signals for nuclei (DAPI); red for endothelial cells (Isolectin-B4).

**Fig 6:**
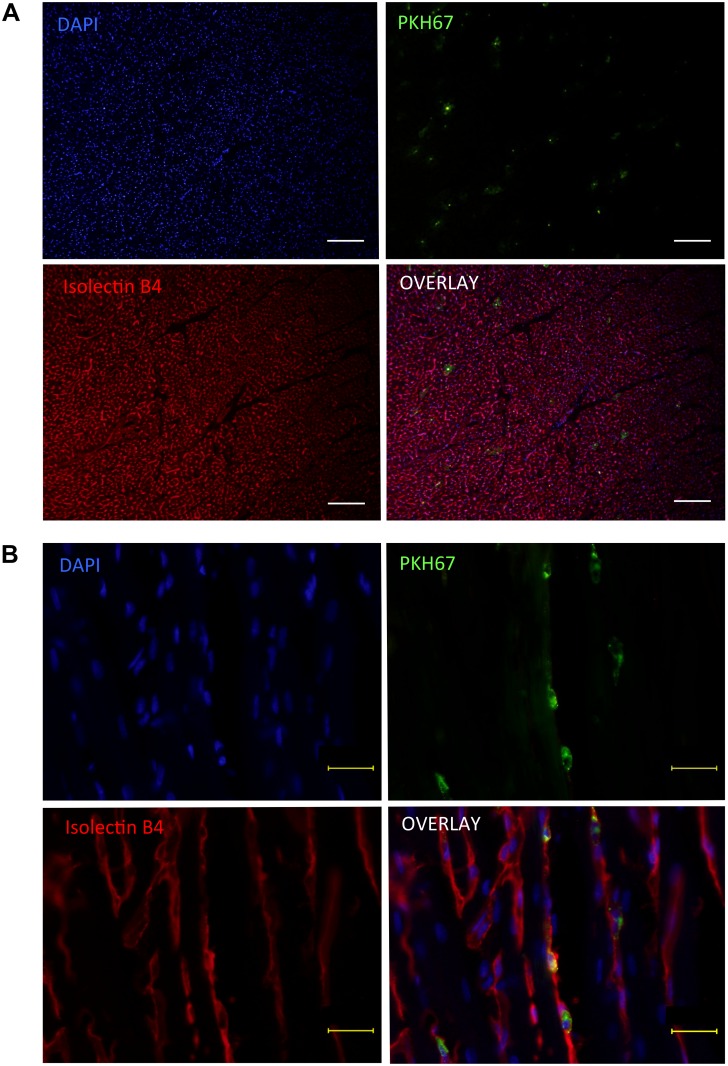
Histological findings of BMMNC retention 60 min after IC injection. Recipient hearts were collected at 60 minutes after IC injection of 8x10^6^ BMMNC that had been labeled stained with PKH67. **A** and **B** represent low- and high-magnification micro images of Isolectin-B4 staining, respectively (scale bar = 100 μm [A] and 30 μm [B]). Representative micro images from n = 4 hearts are presented. Retained BMMNC continued to be localized in the microvasculature in isolation. No donor cell (green) was observed to have extravasated out of the coronary vasculature. Blue signals for nuclei (DAPI); red for endothelial cells (Isolectin-B4).

The location of retained BMMNC in relation to the coronary vasculature, either within or outside the vascular cavity (representing endothelial adhesion/entrapment or extravasation, respectively), was determined using the heart injected with 8x10^6^ PKH67-labelled BMMNC through the IC route. At 5 minutes after IC injection, the observed PKH67-labelled cells were found in isolation and always entrapped inside the vasculature (Fig 5). At 60 minutes after cell injection, donor cell localization remained the same; no observed PKH67-labelled cell have extravasated out of the coronary vasculature (Fig 6). In addition, there were no histological findings to suggest coronary embolism including intravascular cell-clumping.

**Fig 7:**
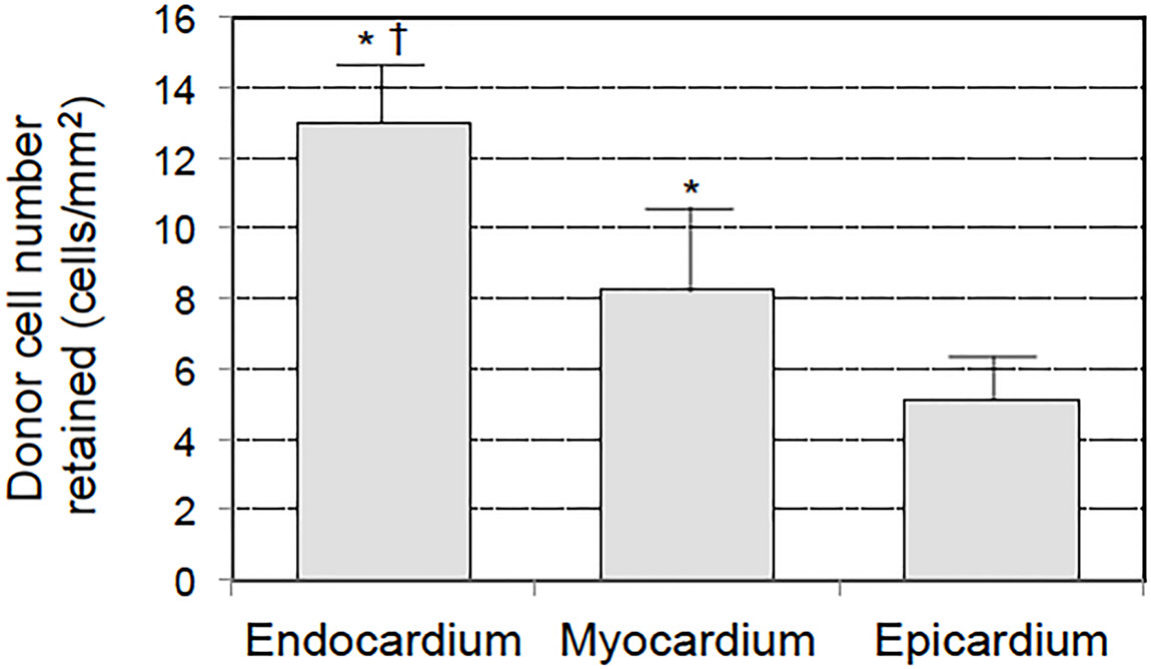
Different distribution of retained BMMNC between ventricular layers. At 5 minutes after IC injection of 8x10^6^ PKH67-labeled BMMNC, the number of donor cells within the endocardium-side myocardium, central myocardium and epicardium-side myocardium were counted in cross-section of immunohistolabelling samples. The concentration of cells progressively increased from the epicardium to the endocardium (n = 4, *p*<0.0001, One-way ANOVA followed by Bonferroni post-hoc test).

At 5 minutes after IC injection of 8x10^6^ BMMNC, the histologically-estimated retention ratio in the whole heart was 18.5±7.7% (n = 4 hearts). In addition, it was observed that the distribution of donor BMMNC was not uniform between the layers of the ventricular wall. There was a progressive increase in concentration of donor cells towards the endocardium (Fig 7). The frequency of donor cells in the endocardium was three times higher compared to that in the epicardium.

### Increased initial retention of MSC in the heart after IC injection

**Fig 8:**
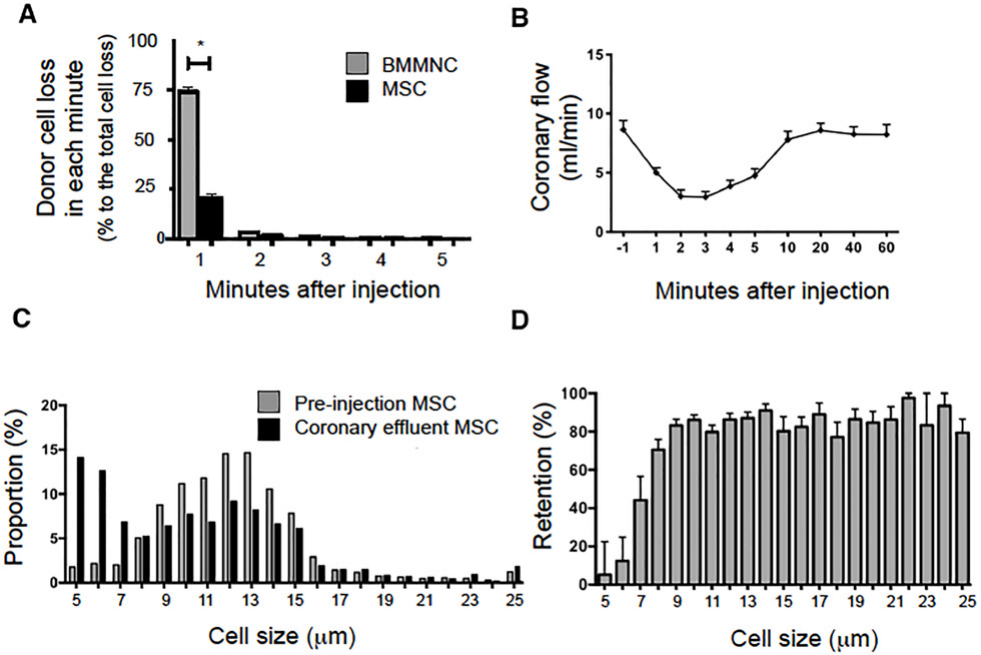
Initial retention of MSC after IC injection. (**A**) Following IC injection of 1×10^6^ bone marrow-derived rat MSC into a rat heart using the same model, reduced numbers of donor MSC were found in the coronary effluent within the first minute, in comparison with IC injection of the same number of BMMNC (refer to Fig 1). n = 8 in each cell-type, *p*<0.001, Two-way ANOVA followed by Bonferroni post-hoc test; *p<0.05 versus Epicardium, ^†^p<0.05 versus Myocardium. (**B**) The coronary effluent flow rate decreased immediately following IC injection of MSC but gradually recovered to the base line by 10 minutes (n = 8). (**C**) The distribution of MSC diameters prior to injection and in the coronary effluent were quantified and expressed as fractions (n = 8). There appeared to be a leftwards shift in the diameters of the coronary effluent cell population. (**D**) With knowledge of the cell numbers of each cell diameter, the retention efficiency of MSC having each diameter was calculated (n = 8). Larger MSC subsets were likely to be more frequently retained, but the retention rate was plateaued at ~80% with cell diameters ≥ 9 μm.

Bone marrow-derived MSC is another preferred donor cell type for cell therapy for heart disease [23,24], which have a larger diameter than BMMNC. To further confirm the positive relationship between cell-size and retention rate, we assessed the initial retention of MSC using the same rat heart *ex-vivo* perfusion model. Within the first minute of IC injection of MSC, it was found that a sizably reduced number of injected cells were flushed out of the heart, compared to IC injection of the same number of BMMNC (Fig 8A). The initial retention of MSC was calculated to be 77.5±1.8%, which was >3-fold higher than that for BMMNC (20.1±3.7%, *p*<0.01). However, the increased donor cell retention of MSC was associated with a temporary reduction in coronary flow (Fig 8B). Immediately after IC injection, mean coronary flow rates dropped to below half of the pre-injection volumes, but gradually recovered until they normalized by 10 minutes after injection.

The median and mean cell sizes of MSC pre-injection were 11.5 and 12.2 μm respectively, while those of the cells in the coronary effluent were markedly reduced; 8.7 and 10.1 μm, respectively (Fig 8C). In addition, as with BMMNC injection, it was found that larger MSC were retained with an increased retention efficiency compared to smaller MSC (Fig 8D). This retention efficiency reached a plateau for cells ≥9 μm in diameter. On the other hand, the retention ratio of MSC sized 5–6 μm were less than 10%.

### Histological assessment of MSC retention in the heart

**Fig 9:**
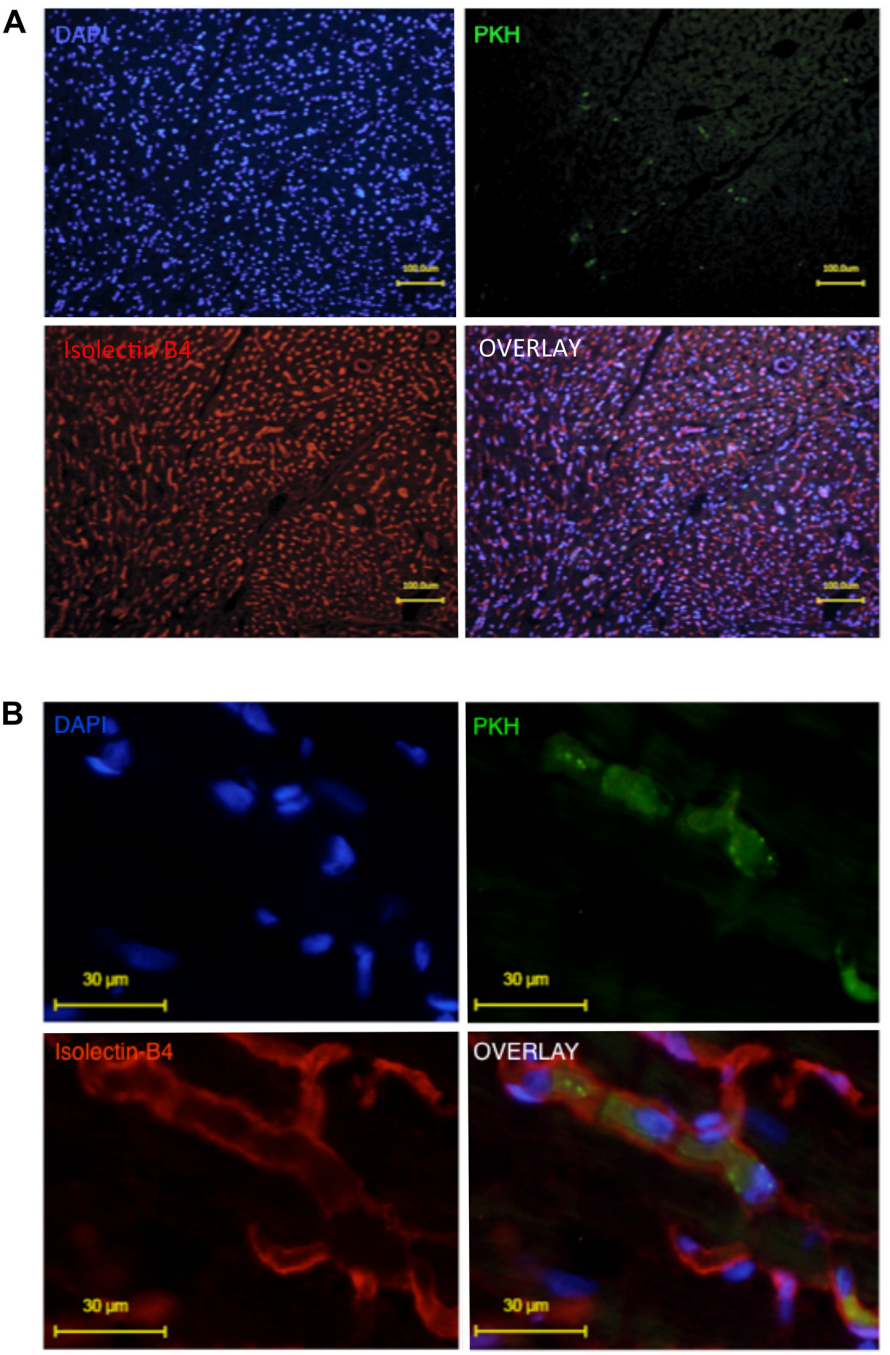
Histological findings of MSC retention after IC injection. Recipient rat hearts were collected at 60 minutes after injection of MSC which had been labeled with PKH67. **A** and **B** represent low- and high-magnification images of isolectin-B4 staining, respectively (scale bar = 100 μm [A] and 30 μm [B]). Representative micro images from n = 4 hearts are presented. All donor cells (green) were found within the coronary vasculature, with frequent formation of cell-clamps in the vasculature. The diameter of vasculatures having MSC appeared to be enlarged, compared to other intact ones. Blue signals for nuclei (DAPI); red for endothelial cells (Isolectin-B4).

After IC injection of PKH67-labelled MSC, the observed donor cells were found always entrapped inside the vasculature; no observed PKH67-labelled cell have extravasated out of the coronary vasculature, similar to BMMNC (Fig 9). Different from BMMNC, it was noted that retained MSC frequently formed cell-clumps in the microvasculature and appeared to enlarge the vascular diameter, suggesting that MSC blocked the coronary micro-vessels. This finding agreed with the reduced coronary flow after IC injection of MSC.

## Discussion

We developed a unique rat model that enabled quantitative minute-by-minute monitoring retention of donor cell in the heart after IC injection. This model also allowed collection and analysis of donor cells that exited the heart. These features of this model facilitated the precise study of factors influencing early donor cell retention after IC injection. The BMMNC retention rate at 5 minutes was poor (~20%) after IC injection into the normal heart, and this proportion was consistent between three different BMMNC doses. MSC, a larger cell type (median diameter 11.5 μm *versus* 7.0 μm for BMMNC), were retained with a markedly enhanced rate (77.5% for MSC *versus* 20.1% for BMMNC). Furthermore, detailed quantitative cell size analysis clarified that larger subpopulations of BMMNC and MSC showed proportionally increased retention rates with a plateau at 9 μm in diameter after IC injection. In contrast, consistently in both cell types, less than 10% of injected cells sized 5–6 μm were retained. These retention data agree with the reported diameter of capillaries. The diameter of coronary capillaries has been reported to be 5.6±1.3 μm in dogs [25] and 5–7 μm in humans [26]. Intravital microscopy work has calculated the diameter of rat cremasteric capillaries to be 7.2±0.3 μm [27]. The diameter of terminal coronary arterioles in rat perfused Langendorff hearts has been shown to be 10–11 μm [28]. Histological analyses suggested that retained cells, either BMMNC or MSC, were entrapped in the microvasculature and did not extravagate at least by 60 minutes after injection. On the other hand, the fraction of donor BMMNC expressing individual cell-surface protein (including integrins, selectin ligand and adhesion molecule) were not altered between pre-injection BMMNC and coronary effluent BMMNC, suggesting that active (biochemical) interactions between donor cells and coronary endothelium are not a critical factor of initial retention of BMMNC after IC injection. All these data consistently indicate that passive (mechanical) intravascular entrapment is the responsible mechanism for initial retention of BMMNC after IC injection with a normal coronary vasculature. The theory that larger cells are more frequently entrapped in the heart after IC injection is simple and easy to accept; however, there has been no appropriate evidence to support it so far. This study provides the first scientific evidence that cell size is a major determinant for the retention of donor BMMNC after IC injection at least in the heart with normal coronary vasculatures.

For assessment of donor cell retention after IC injection, this study applied the *ex-vivo* Langendorff heart perfusion model, which is an established and frequently used technique in cardiovascular research (for other purposes) in rodents. Although some highly experienced laboratories successfully utilize mice in this model, this is technically challenging for most researchers due to the small animals’ size. We previously developed a mouse model to assess donor cell retention [16], and it was now translated to rat in this study, offering wider technical accessibility. The retention rates calculated using the cell numbers in the effluent were well correlated with the retention ratios evaluated using histological cell counts. Both these data are also compatible with the retention rates observed in previous human and large animal studies [19,20], validating the reliability of our mathematical method based on the donor cell number in coronary effluent to assess donor cell retention. Potential limitations of this model may include the lack of blood components (cytokines, growth factors, and blood cells such as neutrophils and platelets) in the perfusate, and that perfusion is non-pulsatile. However, it is important to note that donor cell retention after clinical IC cell injection usually takes place under almost crystalloid conditions, where donor cells suspended in non-blood, serum-free solution/buffer are injected into the coronary artery while occluding its proximal portion with a balloon [1–13]. This stops pulsatile blood flow in the target coronary artery, and the most of remaining blood is likely to be flushed out and replaced with cell suspension.

Results obtained in this study offer several clinical and scientific implications of cell transplantation therapy for heart failure. Firstly, it was observed that injection of BMMNC at 3 different and clinically relevant cell doses did not impact on myocardial perfusion parameters, suggesting that even the highest cell doses studied (40×10^6^ cells in rat representing 1×10^10^ in human subjects) did not cause significant coronary embolism. This adds experimental evidence to confirm the safety of BMMNC transplantation by IC injection. On the other hand, IC injection of MSC markedly reduced coronary flow. Although this change was temporary, extreme caution will be needed when MSC are injected by the IC route, particularly for patients with diseased and stenosed coronary arteries with atherosclerosis. The reason for the recovery of coronary flow may be that some (not all) of temporary-formed aggregations of MSC in the vasculature lumen were gradually loosen, dissolved and flushed out during 60 minutes post IC injection, resulting in recovery of myocardial flow. In addition, there could be increased bypass (collateral) flow and/or dilatation of vasculatures in surrounding areas in response to local ischaemia, compensating the reduced myocardial perfusion. Secondly, initial retention of BMMNC was only 20% after IC injection into the normal heart. These rates might not be applicable when BMMNC are injected into hearts with coronary pathophysiology, such as acute myocardial infarction when endothelial cells are activated and express adhesion molecules. However, we speculate that our findings would be replicated in cases of treatment of non-ischemic cardiomyopathy or post-infarction chronic heart failure, where coronary vessels are relatively normal [29]. Development of strategies to increase initial retention is important to improve the therapeutic effect of IC injection of BMMNC for chronic heart failure. Thirdly, it is likely that poor initial retention also occurs for other donor cell types for stem cell therapy, including cardiac progenitor cells or pluripotent stem cells. It is important to elucidate the initial retention rate and the mechanisms underpinning initial retention of these cell types for their effective future success. Fourthly, larger subpopulations of BMMNC are preferentially retained in the heart. This may be relevant to the findings of other reports showing that a certain subpopulation of BMMNC play a role in therapeutic effects induced by BMMNC transplantation [30, 31]. What subset(s) is the main contributor to the therapeutic effects of BMMNC transplantation (i.e. small or large subset?) remains an unanswered question. The smaller cells may be more likely to be stem/progenitor cells, while the larger cells, including MSC, also have strong potential to induce myocardial repair by the secretion-mediated paracrine effects. Further investigations to dissect the role of small and large subsets of BMMNC in the BMMNC-based therapy as well as development of a method to improve retention of each subset are interesting and important for further success of the BMMNC transplantation therapy.

Base on our data demonstrating that there is no difference in surface markers phenotypes between pre-IC BMMNC and BMMNC of the coronary effluent, it is likely that the retained BMMNC show different cell surface makers expression. However, we could not provide direct evidence about expression of cell surface markers in really retained BMMNC. At 5 minutes after IC injection of 8x10^6^ PKH67-labelled BMMNC, we digested the heart enzymatically and collected the total heart cell suspension as we previously described [32,33]. Then, PKH67^+^ cells were sorted by using fluorescence-activated cell sorting. However, this method could not yield sufficient numbers of PKH67^+^ cells to perform flow cytometric characterisations (repeated 3 times). This lack of direct characterization of the retained cells may be one of limitations of our study. It will also be useful to add an experiment using blocking antibody in order to further confirm the functional role of a certain adhesion molecules in retention of BMMNC.

In conclusion, by developing a user-friendly rat model based on *ex-vivo* Langendorff heart perfusion, we demonstrated that cell size-dependent, passive (mechanical) intravascular entrapment is responsible for the initial donor cell retention after IC injection of BMMNC into the normal heart. The results obtained provide important implications for optimizing clinical protocols of IC injection of stem or progenitor cells to the heart. In addition, this technique is a useful experimental method for the quantification and qualification of early cell retention after IC cell injection, offering wide application (e.g. determination of retention of other cell types) in future studies.
